# Electro-Mechanical Properties of Metallized Sodium Alginate Foils at the Limit of the Electrical Conduction

**DOI:** 10.1021/acsomega.5c04447

**Published:** 2025-07-18

**Authors:** Cristiano Albonetti, Carlo Gotti, Luca Pasquini, Nicola Gilli, Fabiola Liscio, Angela Longo, Stefano Chiodini, Franco Dinelli, Maria Letizia Focarete, Mirko Seri, Monica Bertoldo, Piera Maccagnani

**Affiliations:** † 9327 Consiglio Nazionale delle Ricerche-Istituto per lo Studio dei Materiali Nanostrutturati (CNR-ISMN), Via Gobetti 101, 40129 Bologna, Italy; ‡ Advanced Mechanics and Materials, Interdepartmental Center for Industrial Research (CIRI-MAM), Alma Mater Studiorum, Università di Bologna, Viale Risorgimento 2, 40136 Bologna, Italy; § Department of Physics and Astronomy “Augusto Righi”, Alma Mater Studiorum Università di Bologna, Viale C. Berti Pichat 6/2, 40127 Bologna, Italy; ∥ Consiglio Nazionale delle Ricerche-Istituto per i Polimeri, Compositi e Biomateriali (CNR-IPCB), Piazzale Enrico Fermi 1, 80055 Portici, Italy; ⊥ Center for Nano Science and Technology (CNST), Fondazione Istituto Italiano di Tecnologia (IIT), Via Rubattino 81, 20134 Milano, Italy; # Consiglio Nazionale delle Ricerche-Istituto Nazionale di Ottica (CNR-INO), Via Gobetti 101, 40129 Bologna, Italy; ∇ Department of Chemistry “Giacomo Ciamician” and INSTM UdR of Bologna, Alma Mater Studiorum Università di Bologna, Via Gobetti 85, 40129 Bologna, Italy; ○ Interdepartmental Center for Industrial Research in Health Sciences and Technologies, Alma Mater Studiorum Università di Bologna, Via Tolara di Sopra, 41/E, 40064 Ozzano Emilia, Bologna, Italy; ◆ Department of Chemical, Pharmaceutical and Agricultural Sciences, 9299 University of Ferrara, Via L. Borsari 46, 44121 Ferrara, Italy

## Abstract

In recent years, much attention has been given to biopolymers and renewable raw materials obtained from nature to find alternatives to petroleum-based materials. In this context, we developed a free-standing and flexible conductive substrate by sputtering a thin layer of gold onto a foil of sodium alginate, producing conductive self-standing substrates. These substrates have been utilized for the fabrication of eco-designed solution-processed optoelectronic devices. Herein, we report experimental work to study the mechanism behind the dependence of electrical resistance on the mechanical deformation. Data obtained from mechanical measurements, such as strain, stress, deformation, and bending, are correlated with morphological (Atomic Force Microscopy and Transmission Electron Microscopy) and structural (X-ray Diffraction) data relative to both the surface and the subsurface regions of the metallized substrates. Collectively, these data enabled the elucidation of both the composition and spatial distribution of the metal clusters implanted within the polymer matrix. The substrates present an anisotropic Young modulus, making them more stretchable in-plane with respect to out-of-plane. In the elastic regime, the reproducibility of the electrical resistance variations with respect to the stress applied makes these substrates robust candidates for the realization of strain sensors.

## Graphical abstract

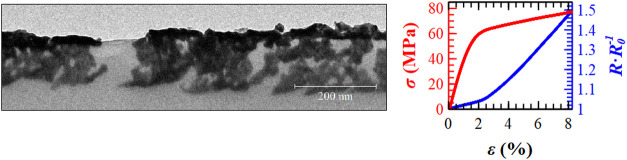

## I Introduction

Lately, driven by the aim to find alternatives to petroleum-based materials, there has been a growing interest in biodegradable materials, derived from either synthetic or natural sources.^1^ In particular, significant attention has been given to biopolymers and renewable raw materials obtained from nature, such as bacterial cellulose,^2^ silk fibroin naturally produced by larvae,^3^ and sodium alginate, a natural anionic biopolymer obtained from marine algae.^4^ These materials exhibit several desirable characteristics: their production by nature contributes to CO_2_ sequestration, they possess good mechanical properties, and they are suitable for a wide range of applications, such as edible films, food packaging, and biomedical devices.^5^

Within this context, in recent years, we have developed a free-standing and flexible conductive substrate by sputtering a thin layer of gold onto a foil of sodium alginate. We have thus created conductive foil approximately 110 μm thick, with a surface conductivity of 20 Ω/sq and a surface roughness of 2 nm.^6,7^ These substrates have been utilized for the fabrication of eco-designed solution-processed organic solar cells^6^ and organic light emitting diodes,^8^ although they have potential applications in numerous fields, including bioelectronics, wearable devices and sensors, the Internet of Things, and more.

Based on previous experiments^9–11^ and data of metal clusters deposited on other polymeric substrates reported in the literature,^11–13^ we have planned to further investigate the electrical resistance at the conduction limit.^14^ In particular, the mechanisms behind the resistance dependence on mechanical deformations are critical in view of electrical conduction through metal films deposited on polymer films. Herein, we report data based on the use of tensile, hardness, and compressive testing machines to measure strain and stress, deformation, and bending, respectively. In addition, Atomic Force Microscopy, X-ray diffraction, and Transmission Electron Microscopy have been employed to determine the morphology and the structure of both surface and subsurface regions of the metallized sodium alginate-based substrates.

## II Materials and Experimental Methods

### II.I Fabrication of Sodium Alginate Thin Disks

Sodium alginate (SA) foils were produced by pouring 26.8 g of a 4 wt % solution into a 100 mm polystyrene Petri dish. The solution is obtained by dissolving an alginic acid sodium salt (Sigma–Aldrich) in ultrapure water at room temperature (RT). Prior to casting, the Petri dish is cleaned by rinsing several times with isopropanol and dried with nitrogen flux. Once cast, the solution was left drying for 10 days in clean room air (ISO8 classification). After that, the SA foils were manually detached, obtaining transparent freestanding disks with a homogeneous thickness of (150 ± 20) μm. The final disk diameter is 90 mm, with the exclusion of the external annular region. All the SA disks (SAD) herein employed were produced in the same ambient conditions with a temperature T of (21 ± 2) °C and a relative humidity RH of (65 ± 4) %, thus avoiding chemical or physical changes due to RH or T variations.^15,16^

### II.II Metallization

The SAD side in contact with the Petri dish was metallized by sputtering gold (MRC 8622 RF system, Kenosistec s.r.l., Binasco, Milano, Italy). Prior to Au deposition, the system chamber is evacuated to a base pressure of 3.2 × 10^–8^ mbar that, after injecting 70 sccm of Ar, raises to 1.9 × 10^–3^ mBar. Sputtering is performed with a frequency of 13.56 MHz by applying a self-bias voltage of −453 V and a power of 20 W, corresponding to an electrical current of ≈4.4 × 10^–2^ A.^17^ At 20 W, the Au target (99.999% pure) bombarded with Ar^+^ ions produces nanometric clusters of different size and crystal orientation,^18^ escaping from the target with an average energy of (280 ± 50) eV.^19^ During the sputtering deposition, the substrate holder is placed 10 cm apart from the target, grounded, and kept at RT.^20^ The plasma is confined close to the target to avoid affecting the substrate surface. The deposition rate *R* and the thickness of the Au films *h*_0_ were calibrated by depositing Au clusters on Si wafers (see detailed procedure in Supporting Information). In agreement with ref 21, a rate *R* of (0.15 ± 0.01) nm·s^–1^ corresponds to a film thickness *h*_0_ ≈ 22 nm for a deposition time of 160 s. This is the minimum time required to obtain conductive SADs with reproducible resistance values.^9–11^

### II.III Topographic Imaging with Atomic Force Microscopy

The morphologies of the two SAD sides were characterized by Atomic Force Microscopy (AFM) operating in intermittent contact or in contact mode. All the topographic images were recorded with a standalone system (HV-SMENA NT-MDT, Moscow, Russia) in ambient conditions, employing silicon cantilevers with resonant frequencies from 180 to 380 kHz and a nominal tip curvature radius of 10 nm (Scout 350, NuNano, Bristol, U.K. and HA_NC, NSG01, NSG11, NSG11/Pt, CSG01, NT-MDT, Moscow, Russia). The images were processed with the software Gwyddion.^22^

Phase data were simultaneously recorded with the topographic ones to maintain the tip–sample interaction either in the attractive or the repulsive regime.^23^ Out of contact, the phase ϕ of the free cantilever oscillations is 90°, with a delay with respect to the sinusoidal signal driving the cantilever oscillation. When the tip interacts with the surface, the cantilever dissipates part of its energy due to nonconservative tip–sample interactions, and a phase lag Δϕ in the cantilever oscillations occurs.^24^ The phase ϕ is thus (90° + Δϕ), with ϕ > 90° in the attractive regime and ϕ < 90° in the repulsive regime (as established by Cleveland et al.).^24^ In the repulsive regime, the tip indents the surface and the overall derivative of the forces exercised by the tip to the sample can be approximated to the local sample stiffness *S*.^25^ Assuming a force derivative very small in magnitude compared with the cantilever elastic constant *k*,^26^ an approximation which holds true in our case, the phase lag Δϕ (in rad) is related to the local stiffness *S* through the relationship:

1 S=k·Δϕ/Q

where *Q* is the cantilever quality factor. Equation 1 shows that phase imaging provides a map of the stiffness variation on the sample surface, so that regions with larger Δϕ values are stiffer. The surface roughness *R*_a_ and the Autocorrelation Function (ACF) were used as mathematical descriptors of the surface morphology.^27,28^

### II.IV Grazing Incidence X-ray Diffraction Measurements

Grazing Incidence X-ray Diffraction (GIXRD) measurements were conducted at the MCX beamline of the ELETTRA synchrotron using an X-ray energy of 10 keV, corresponding to a wavelength of 1.24 Å. The incident angle ω can be varied from 0.4 to 2° to investigate the surface with a different penetration depth Ξ. For an ideal continuous thick layer of Au or SA, Ξ varies from approximately 3 nm (10 μm) to over 100 nm (56 μm). Prior to X-ray measurements, the samples were topographically imaged with AFM to identify surface patterns and orient them parallel to the X-ray beam. This expedient reduces the additional beam scattering induced by morphological features.

### II.V Transmission Electron Microscopy Measurements

The vertical structure of the metalized SADs can be investigated by Transmission Electron Microscopy (TEM). The subsurface region is transferred onto an ultrahigh mesh Nickel foil shaped over a 200 mesh TEM Cu grid with the solvent-assisted transfer-printing technique.^29^ TEM measurements were performed with a FEI Tecnai F20 ST TEM, operating with a nominal accelerating voltage of 200 kV, equipped with an energy dispersive X-ray system (EDS). Noninvasive measurements of the subsurface region were obtained from the analysis of cross-sectional slices (100–150 nm thick), produced by cutting samples of SADs embedded in epoxy resin (Epon 812), with a Leica UC6 ultramicrotome at RT. Dry sectioning was performed to obtain slices, which were then deposited on a carbon-coated copper grid. Bright field TEM images of the slices were acquired using a Talos L120C system (Thermo Fisher Scientific), operating at 120 kV and equipped with LaB_6_ illumination.

### II.VI Electro-Mechanical Characterizations

Full mechanical and electro-mechanical characterization of pristine and metallized SADs were performed using tensile, nanoindentation, and compressive testing machines.

Tensile tests were performed with an Instron testing machine (Model 4465, Norwood) equipped with a ±100 N load cell. The samples were rectangular stripes 50 mm × 5 mm obtained by cutting pristine and metallized SADs with a die-cutting machine and a manual press. The sample holder setup was specifically designed to allow accurate, precise, and reproducible positioning of the rectangular specimens between the machine grips (Figure 1). Such a setup prevents sample slippage and, concurrently, grants good electrical contact. A dedicated experimental setup was devised to reproducibly replicate the same experimental conditions. The sample is first mounted in a paper frame (outer dimensions 50 mm × 25 mm, inner dimensions 26 mm × 12 mm) by using double tape. Two thin, striped electrodes made of copper tape were placed at the ends of the inner window of the paper frame in contact with the SAD metallized side. To prevent slippage, the abrasive part of two sandpaper pieces (grit 400) was used to sandwich the sample and electrodes, adhering to them through the double tape. The external paper segments were cut before starting the experiment, resulting in an effective length of 26 mm between the clamps. Electrical resistance measurements on conductive samples were performed with an LCR meter (Figure 1, LCR-6200, GW Instek, Montclair), operating in Direct Current Resistance (DCR) mode.

**Figure 1:**
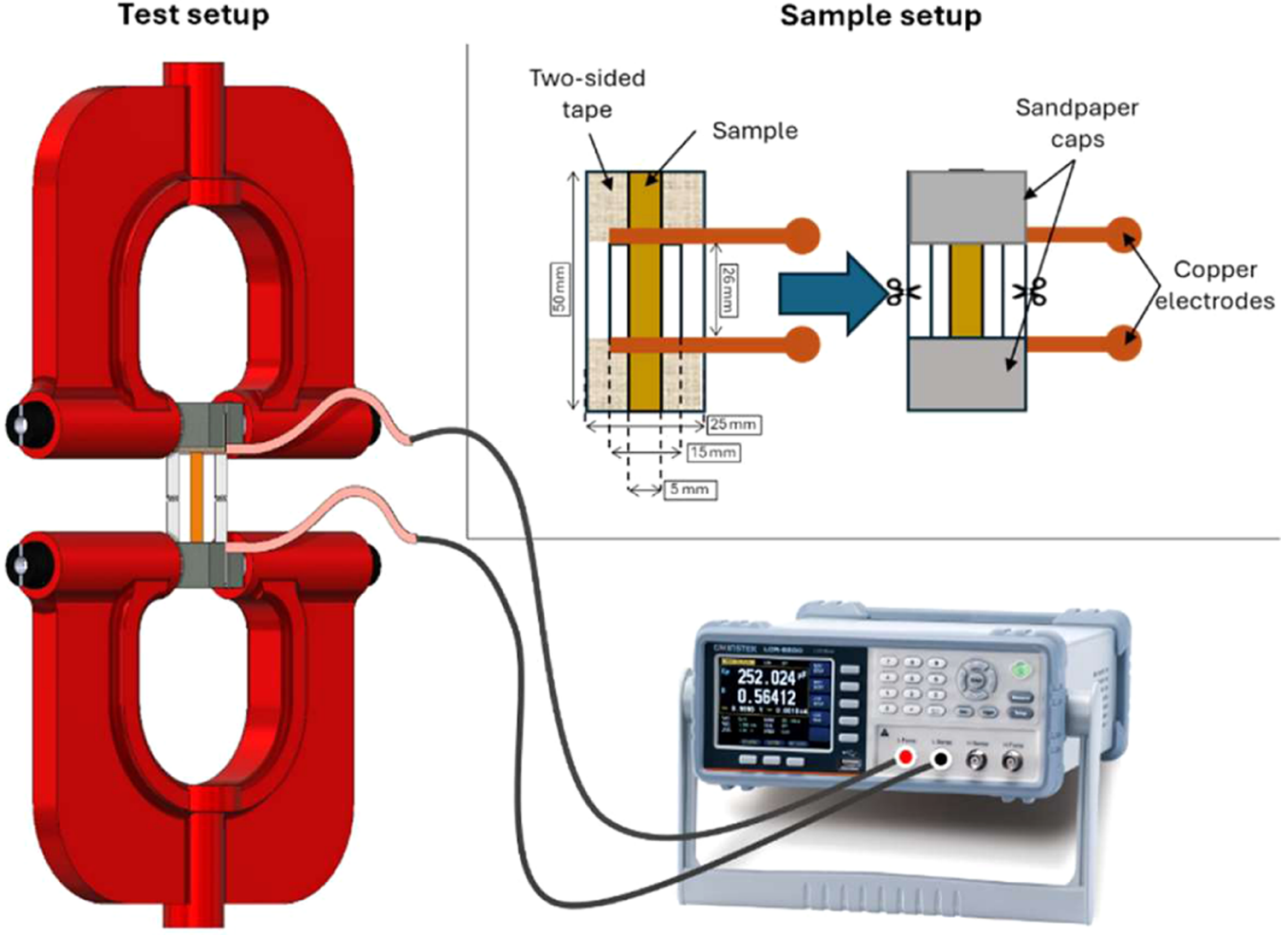
Experimental setup for electro-mechanical tests: samples consisting of 50 mm × 25 mm stripes were fixed to a paper frame through double tape and sandpaper. Two copper streaks were embedded as electrodes. The samples were thus clamped in an INSTRON 4465: the electrical resistance was measured during tensile tests.

Two types of mechanical tests were performed. (1) Failure Tests: They are conducted under displacement control with a monotonic ramp up to failure at a 0.5 mm min^–1^ rate. The apparent stress was calculated by dividing the measured force by the cross-sectional area of the sample. A mechanical characterization of SADs includes stress (σ_F_) and strain (ε_F_) at failure, yield stress (σ_Y_) and strain (ε_Y_), and elastic modulus in tensile tests (*E*_TT_). Such measurements were performed on 15 samples of pristine SADs (P subscript) and 10 samples of metalized SADs (M subscript). The experimental data reported in the manuscript represent the average value and the standard deviation of data sets recorded for pristine and metallized SADs. The electrical resistance *R* of the conductive samples was measured from the unstressed condition up to failure.

(2) Cyclic Tests: Based on the failure tests, two strain levels were identified in the middle (ε = 0.5%) and at the end of the elastic range (ε = 1%). For each strain level, 10 cycles were performed until complete unloading. Cyclic tests were performed on 6 samples of metallized SADs, measuring *R* during each cycle. Data analysis was carried out with a homemade software developed in MATLAB,^30^ previously adopted to estimate the Young Modulus of nanofibers.^31^

Instrumented indentation tests of mechanical hardness and elastic modulus were carried out with a CSM NHT-TTX Nanoindenter equipped with a Berkovich diamond tip. The indentation force *F*_I_ vs the penetration depth Δ was registered up to a maximum force of 0.5 mN, reached with a linear increment of 1 mN·min^–1^. The indentation force was kept constant at the maximum value for 1 s, after which it was decreased linearly at −1 mN·min^–1^. Such parameters correspond to a maximum penetration depth of about 240 nm, as measured by the capacitive displacement sensor of the instrument. The sampling rate was set at 10 Hz. Two square samples of 15 mm × 15 mm of pristine and metallized SADs were fixed by Araldite on two iron parallelograms of 20 mm × 20 mm × 10 mm. A total of 50 measurements were performed on each sample with a minimum distance of 50 μm between two consecutive tests.

The Young modulus *E*_IT_ and the hardness *H*_IT_ obtained from the indentation tests were determined from the Oliver–Pharr analysis of the load–displacement curves. The Young modulus *E*_IT_ corresponds to elastic stiffness, i.e., the ratio between applied stress and elastic strain in the linear part of a macroscopic tensile or compressive test, well below the yield stress. The hardness *H*_IT_ represents the resistance to plastic deformation. For a homogeneous sample, its value can be considered a good indicator of the yield stress observed in a tensile or compressive test or of the classical microhardness determined by optical analysis.^32^

A homemade mechanical buckling system was used to evaluate the radius of curvature (ROC) in the middle of the stripe, produced by compressing rectangular stripes (≈30 mm × 10 mm) of metallized SADs.^33^ The stripe is blocked by two conductive clamps electrically connected with a dual-channel system source meter (Keithley 2602, Beaverton, OR). An endless screw moves one clamp toward the other one fixed, and thus the stripe is bent. I/V curves (in the voltage range from −1 to +1 V) were acquired for different bending values, 10 in total, considering forward and backward, and then *R* and ROC were calculated. The experiments were performed on 5 different metallized samples.

## III Results

Pristine and metalized SADs were characterized both morphologically and structurally to determine how the deposited Au clusters modify the surface and the subsurface region. These characterizations are pivotal for interpreting the electro-mechanical behavior of the metallized SADs.

### III.I Morphology of Pristine and Metallized Surfaces

As shown in Figure 2, the two sides of a pristine SAD, in contact with a Petri dish or exposed to air, have completely different morphologies. The side exposed to air resembles an amorphous polymeric film with a *R*_a_ = (41 ± 4) nm (Figure 2a), while that exposed to the Petri dish presents a pattern of *quasi*-regular threads with a lower *R*_a_ = (7.7 ± 1.6) nm (Figure 2b). The reason for that is evident when the Petri dishes and SAD surfaces are compared (Figure 2c,d). The Petri dish is also characterized by relatively linear threads (area delimited by two dashed yellow lines), spaced by irregular and depressed regions (transparent blue trapezoidal area).

**Figure 2:**
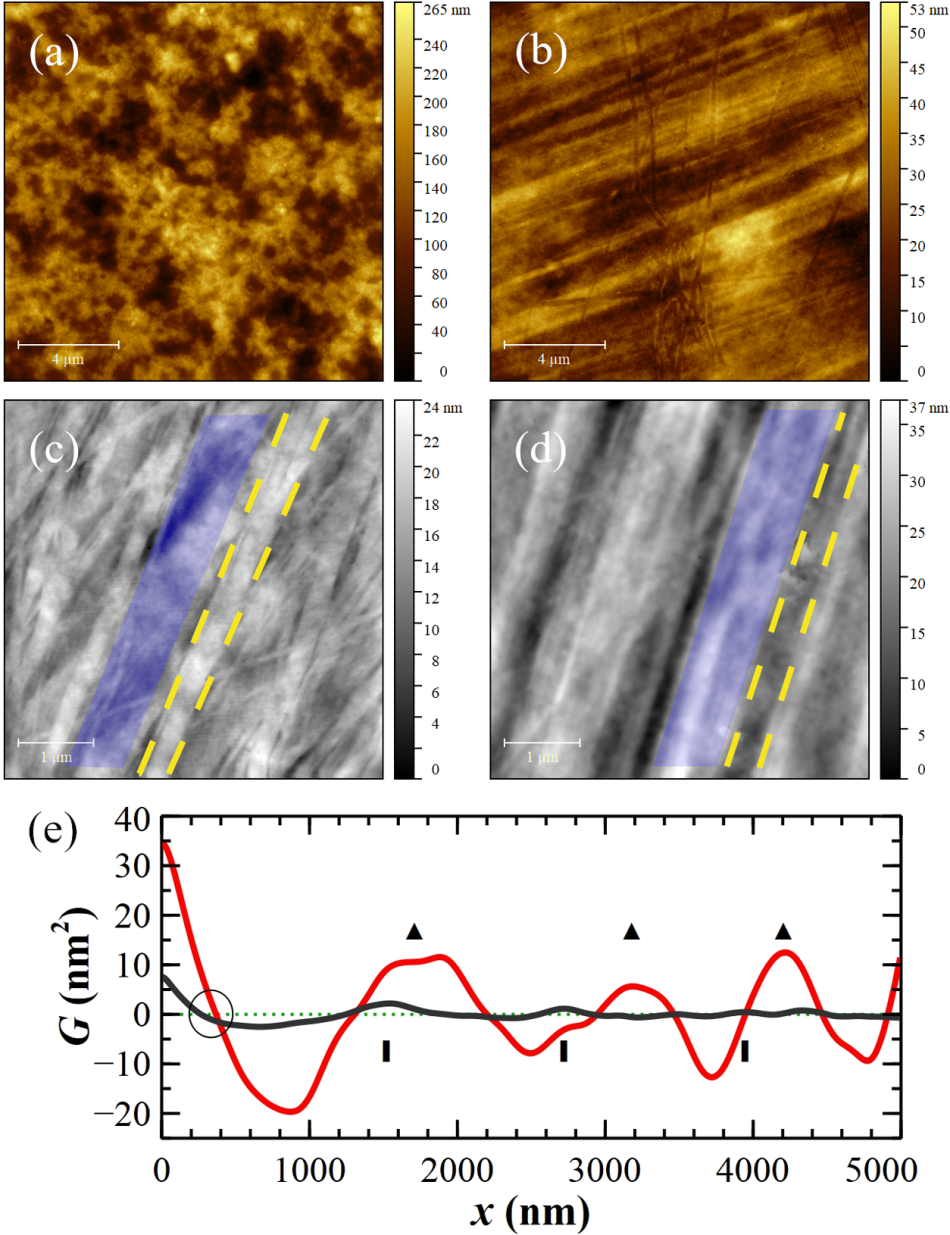
Topographic images 15 × 15 μm^2^ of the SAD side exposed to air (a) and in contact with the Petri dish (b). Higher magnification images 5 × 5 μm^2^ of the Petri dish surface (c) and the SAD side in contact with it (d). The Petri dish is characterized by linear threads (in the middle of the yellow dashed lines) and disordered regions (transparent blue trapezoid areas) that are negatively transferred to the SAD surface. (e) ACF calculated from the AFM images of the Petri dish surface (black line) and the SAD side in contact with it (red line). Rectangles and triangles point at periodic features, while the black circle indicates the first zero-crossing point of the two ACFs.

Since casting works as the replica molding technique,^34^ the SAD surface in contact with the Petri dish is a negative replica of it: flat depressed trenches are spaced by irregular regions in relief (Figure 2d). This is confirmed by the Autocorrelation Function (ACF) of the AFM images: *G* follows a trend due to a mixing of ordered- and slightly ordered- morphological features distributed on the surface (see Figure 2e).^35^ Both surfaces show periodic oscillations with an average distance of ≈1.2 μm (the average separation between rectangles and triangles, respectively). Moreover, the first zero-crossing of the Petri dish ACF is anticipated with respect to the SAD one since its *R*_a_ = (3.1 ± 0.4) nm is about the half (black circle in Figure 2e). The larger roughness observed for the SAD surface is likely attributable to molecular reorganization occurring during the drying process.^36^

All pristine SADs fabricated for these experiments have the same *R*_a_ value (8 ± 1) nm and *quasi*-regular linear patterns, although they are randomly orientated with respect to the radial direction of the disk in different surface regions (see Supporting Information).

At higher magnifications, i.e., 2 × 2 μm^2^, the SAD surfaces show small grains with an average diameter of ≈80 nm, whereas the phase images, in the attractive and repulsive regimes, present a homogeneous contrast. From eq 1, the average stiffness *S* of the pristine SAD surfaces can be evaluated in (1.9 ± 0.4) × 10^–2^ N·m^–1^.

The deposition of the Au layer of 8 nm (nominal) by plasma sputtering leaves the polymeric morphology unaltered both in terms of *R*_a_, (8 ± 1) nm, and in *quasi*-regular linear patterns (compare Figure 3a, pristine, and Figure 3b, Au sputtered). This qualitative evidence excludes aggregation phenomena of the Au clusters, otherwise the *quasi*-regular threads would be smoothed,^37^ or the Au aggregates should appear in relief.^38,39^ The Au clusters are thus expected to be implanted in the subsurface region, in agreement with the literature of plasma deposition on polymers.^40^ To investigate the implanted clusters, phase imaging in the repulsive regime can be employed as it enables to observe subsurface features.^41–43^

**Figure 3:**
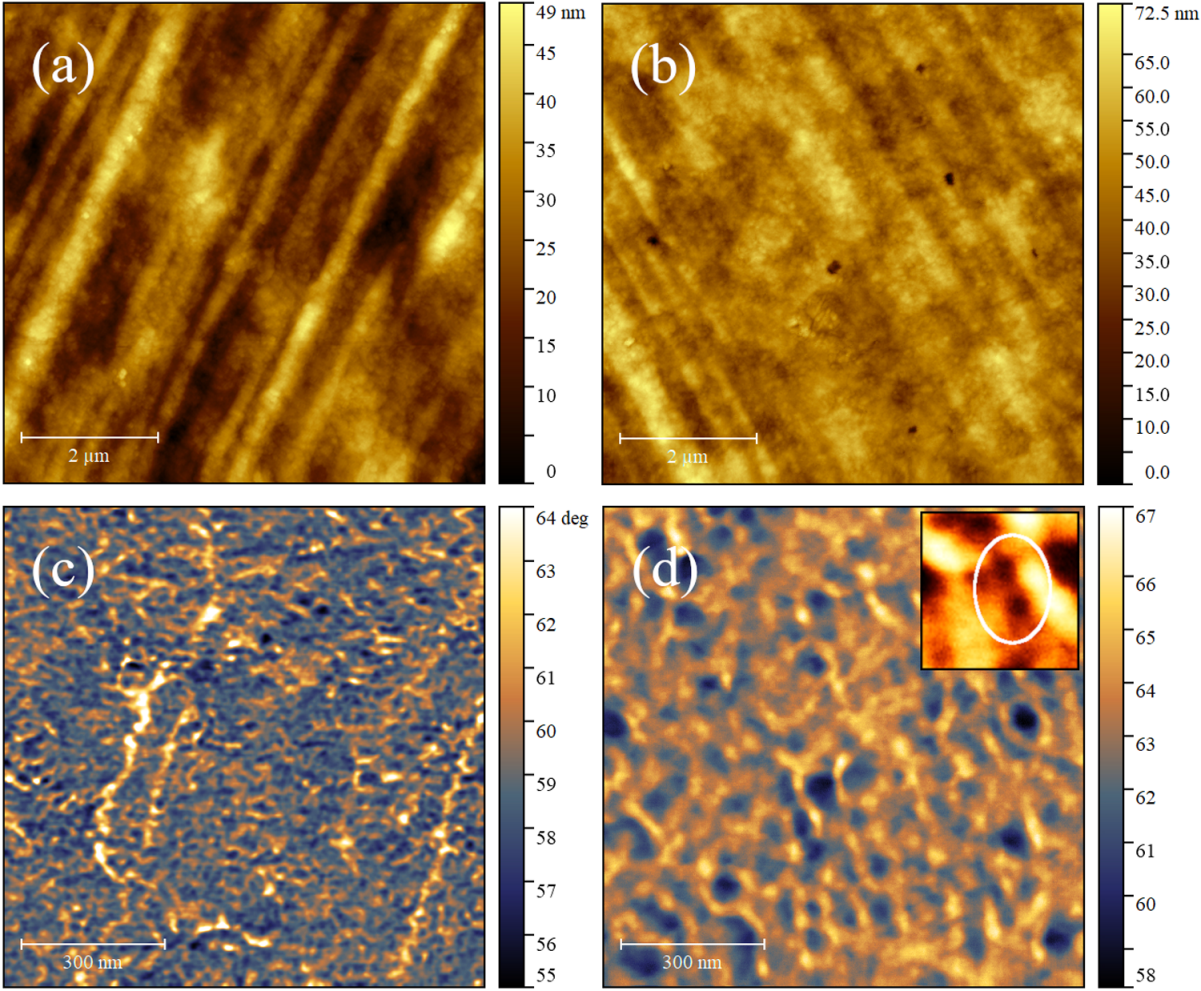
Topographic AFM images 7 × 7 μm^2^ of pristine (a) and metalized (b) SAD surfaces. Corresponding phase images at higher magnifications, 1 × 1 μm^2^, obtained in the repulsive regime on pristine (c) and metallized (d) SAD surfaces with an average tip–sample force of ≈200 pN and 1.3 nN, respectively. Inset: digital zoom ≈140 × 140 nm^2^ to better emphasize the dimensions of the dark spots governing the diameter distribution (circled).

On a pristine SAD surface (Figure 3c), the phase contrast is homogeneous and compact, whereas the Au clusters modify mechanically the surface as shown by the appearance of dark spots surrounded by a bright region matrix, i.e., stiffer areas within a less stiff region. In particular, Figure 3d reminds of a “bombardment”,^40^ showing “phase craters” due to the implantation of clusters. The average diameters of these “craters” are measured by masking the phase image with the inverted thresholding tool and then producing the distribution of the radii of discs with the same projected area as grains.^44^ The distribution is characterized by two peaks at (12 ± 8) and (38 ± 4) nm; the latter, correspondent to a diameter of ≈80 nm, is related to larger dark area visible in the phase image of Figure 3d whereas the former, ≈25 nm, are related to small dark spots circled in the inset of Figure 3d.

The phase contrast of the craters is due to the different mechanical properties of SA and Au, but it may also depend on the implantation depth of the clusters.^45^ Consequently, their average diameter of (12 ± 8) nm may not correspond to the actual diameter.

To further study the cluster dimensions, a deposition was performed on Highly Oriented Pyrolytic Graphite (HOPG) under the same experimental conditions adopted for the metallization of SADs, but with a deposition time of only 5 s (see Supporting Information). Such a short time produces well-separated clusters (surface density ≈40 clusters per μm^2^), thus excluding surface reorganization phenomena. In addition, HOPG is harder than SA, and the clusters do not penetrate it (Young modulus *E* is in the range 20–60 GPa for HOPG and 0.4–0.6 GPa for SA).^46–48^

Assuming a spherical shape, the height measured with AFM is equal to the cluster diameter.^49^ Considering a set of 10 clusters, four height values are found: 2 nm (3 clusters), 3 nm (4), 5 nm (2), and 10 nm (1). The atomic flatness of HOPG also allows to observe the roughening effect due to the Ar^+^ bombardment during plasma deposition:^50^ *R*_a_ increases from ≈0.5 for freshly cleaved HOPG to ≈2 Å for a metallized one. This effect should be more prominent on SA that is much softer than HOPG, with a possible involvement of a chemical and/or physical alteration of the subsurface region.^40,50^

### III.II GIXRD Characterization of the Au Cluster Depth

GIXRD was employed to investigate the structural properties of the metallized SAD surface, accessing various penetration depth (Ξ) values by varying the grazing incident angle ω of the X-ray beam. The ratio between the intensities of Au and SA peaks is a hallmark of the structural composition of the composite at nanoscale depths.

As shown in Figure 4a, a GIXRD scan performed at a very low grazing angle (ω = 0.4°) detects the presence of both Au and SA. The width of the Au peak can be used to estimate the average cluster diameter, around 5 nm.^51^ If there was a continuous layer of Au at ω = 0.4°, one would estimate a Ξ of ≈3 nm, lower than the average diameter of the clusters.^52^ The detection of an SA peak therefore suggests that the Au film does not cover the surface homogeneously and allows the X-ray beam to penetrate through gaps in the Au layer to reach the alginate substrate beneath.

**Figure 4:**
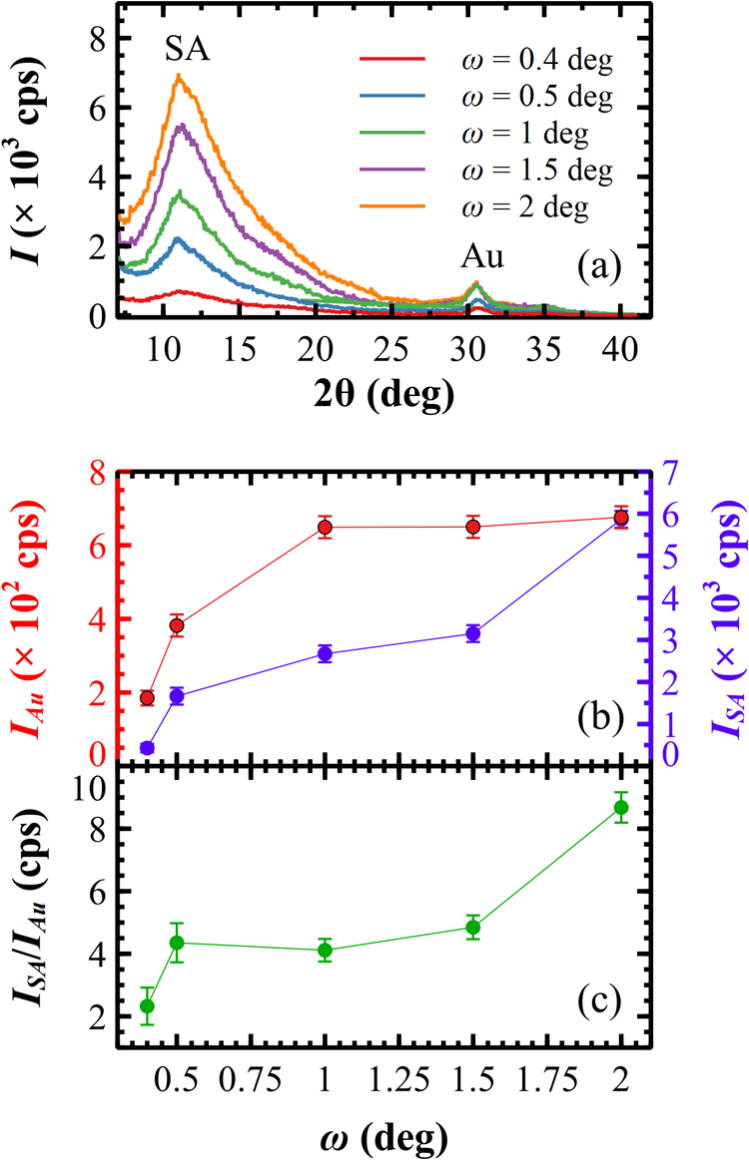
(a) GIXRD patterns for the metallized SAD surface, collected at different incident angle ω values. Below, Au (*I*_Au_) and SA (*I*_SA_) peak intensities (b) and their ratio (c) are plotted vs ω.

At ω = 0.5°, the signal *I*_Au_ increases slightly, whereas the signal *I*_SA_ tripled (Figure 4b). Such a significant increase indicates a greater exposure of SA to the X-ray beam, confirming the nonuniform presence of Au clusters. The doubling of the intensity ratio *I*_SA/Au_ (see Figure 4c) implies that the X-ray beam penetrates a larger volume of SA with respect to Au, indicating a decrease in Au concentration with increasing Ξ.

At ω = 1°, *I*_SA/Au_ remains equal to the value obtained at 0.5°, confirming that at a deeper X-ray penetration, one still detects both SA and Au. The presence of SA hampers a precise determination of Ξ because the material density is variable but, at the same time, confirms that plasma sputtering implants Au clusters deep into SA. As *I*_Au_ increases, some clusters are present even at Ξ≈70 nm, which is the expected penetration Ξ at this angle in the case of a continuous Au layer.

At ω = 2°, *I*_SA/Au_ increases consistently: A deeper X-ray penetration determines a higher detection of SA. However, the Au peak *I*_Au_ remains constant, meaning that the beam does not detect any additional cluster.

The variation of *I*_SA/Au_ and Ξ at different ω evidence the complexity of a composite structure made of Au clusters and SA. This depth profiling suggests that Au clusters are not uniformly distributed, but they are dispersed within the SA matrix. With X-ray and AFM data, we can deduce a picture of a metallized surface composed of Au clusters implanted and not uniformly dispersed in a 3D SA matrix, i.e., they are nonuniformly distributed on the surface plane and down to a depth of ≈100 nm from the surface. This picture is consistent with models of metal diffusion in a polymeric film.^53^

### III.III Cross Section of a Metallized Surface

As reported in the literature, metal clusters deposited on polymeric films using plasma deposition can be both implanted or not implanted.^54–57^ When implanted, they can diffuse within the polymeric matrix down to a depth of ≈100 nm, even at RT,^56^ like in our case.

The interaction of plasma constituents with the polymeric surface leads to its modification,^58^ affecting also the subsurface region with structural and/or chemical changes like breaking of polymer chains, depolymerization, formation of polymer fragments, and cross-linking.^59^ Such a kind of changes increases the solubility of the subsurface region and promotes the plastic deformation of the polymeric substrate,^60,61^ even though its hardness may be increased by the presence of the clusters.^61^

The subsurface region of the metallized SAD surfaces can be transferred onto a flat and rigid substrate with solvent-assisted transfer-printing,^29^ allowing to investigate some components of their vertical structure. A drop of water (≈1.5 μL) was dispensed by a syringe (25 μL Gastight Luer Lock syringe, Hamilton, Bonaduz, CH) on a flat borosilicate glass coverslip (surface roughness of ≈1 nm, Schott D263, Vemi S.r.l., Milano, IT; Figure 5a). A square chip (≈1 × 1 cm^2^) of a metallized surface was placed on the drop (Figure 5b), and then it was gently removed after ≈5 s.

**Figure 5:**
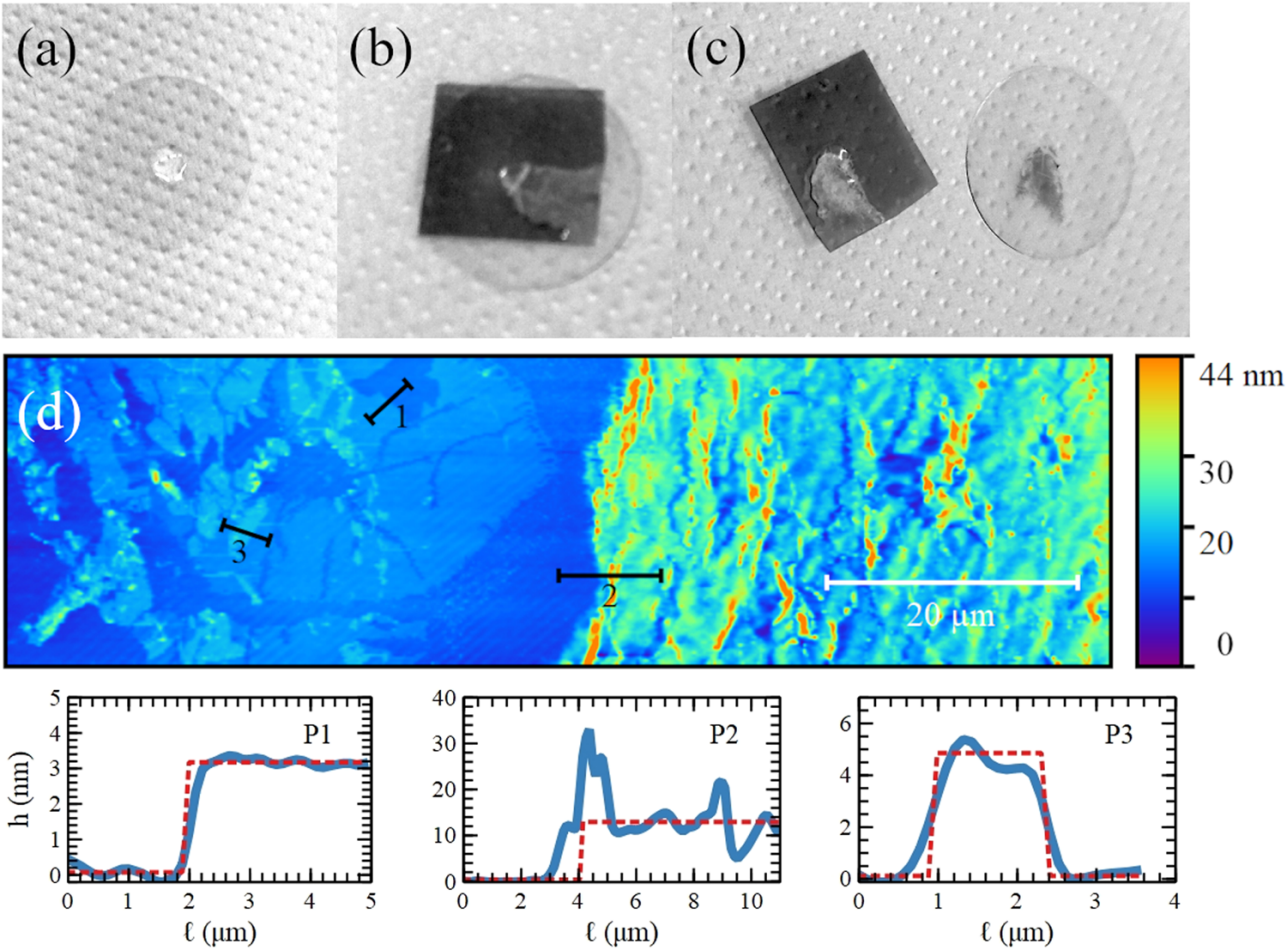
Gray-scale pictures of the solvent-assisted transfer-printing technique applied to a metallized SAD surface. A water drop is dispensed on a circular coverslip (a), and a piece of the metallized SA surface is placed on it (b). The piece of the metallized surface is then gently removed, leaving residual material on the coverslip (c). AFM topographic image of the region transferred onto the coverslip in contact mode (d). Below, the height profiles of three steps are indicated with black segments in the AFM image.

As clearly visible in Figure 5c, this is sufficient time to transfer part of the subsurface region, which becomes transparent in correspondence to the region transferred on the coverslip, confirming the higher solubility of the subsurface region interacting with the plasma constituents. As a countercheck, the same experiment was performed on pristine SADs to obtain a clean coverslip.

The AFM topographic images of the transferred region reveal several structures: flat, ordered, and ultrathin islands (on the left in Figure 5d) and compact, thin but rougher areas (on the right in Figure 5d). Their thickness *h* suggests that these ultrathin islands are composed of Au clusters (profiles P1 and P3 at the bottom of Figure 5) with *h* from ≈3 to ≈5, as obtained on Au clusters deposited on HOPG (see Section 3.1). The region thicker than 5 nm is likely to be a mix of SA and Au (profile P2 at the bottom of Figure 5).

Solvent-assisted transfer-printing techniques often produce thicker films, like the one observed on the right-hand side of Figure 5d. TEM measurements were performed to investigate their structure. The bright field image reported in Figure 6a shows a pronounced material contrast obtained by inserting a diaphragm into the objective lens to exclude electrons with a high scattering angle. These electrons are produced by crystalline elements; therefore, the darker regions of the bright field image are Au clusters, while the wide light gray region is the amorphous SA matrix. On the contrary, the brightest regions are holes in the film.

**Figure 6:**
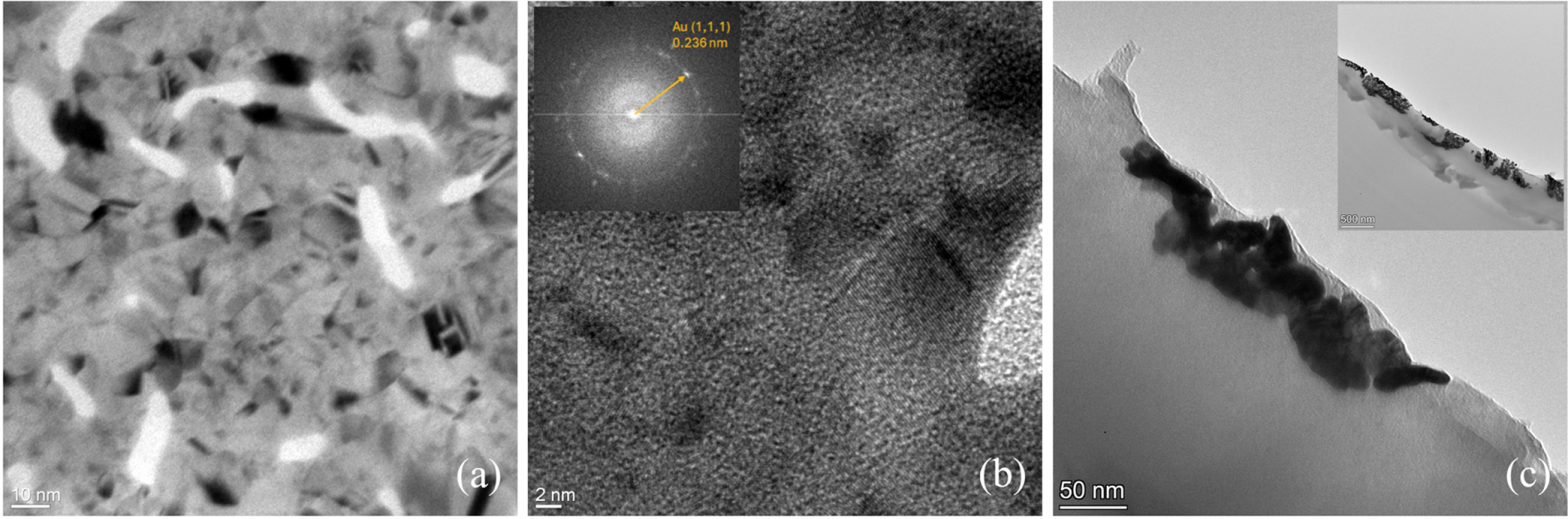
(a) TEM bright field image of the thicker portion obtained with the solvent-assisted transfer-printing technique. (b) High-resolution image of a small region: The diffraction patterns are visible and measured with FFT (inset). (c) Bright field images of a vertical section at a lower (inset) and a higher magnification.

The Au clusters present an irregular shape with a lateral size ranging from ≈3 to ≈15 nm. The darker regions are investigated with high-resolution TEM without a diaphragm (Figure 6b); crystalline domains produce diffraction fringes, as revealed by a Fast Fourier Transform of the entire image (inset of Figure 6b). The diffraction spots in the ring correspond to a 0.236 nm distance, typical of polycrystalline Au(111). This confirms that the darker regions are made of Au.

These measurements allow one to identify the vertical structure components but not the spatial distribution of the clusters. As visible in the inset of Figure 6c, the TEM images of the cross-sectional slices show that the clusters are implanted in the SA matrix within a subsurface region ≈150 nm deep. At a larger magnification (Figure 6c), the clusters can be observed just below the surface, confirming the experimental results obtained with both X-ray diffraction and AFM morphology.

### III.IV Electro-Mechanical Properties of a Metallized Surface

The mechanical properties of both pristine and metallized SADs were measured with tensile and hardness tests. As shown in Figure 7a, stress–strain curves of pristine and metallized SADs show the same trend: an initial linear region associated with elastic deformation, followed by a knee indicating the yield point,^31^ and a subsequent linear segment extending up to failure.

**Figure 7:**
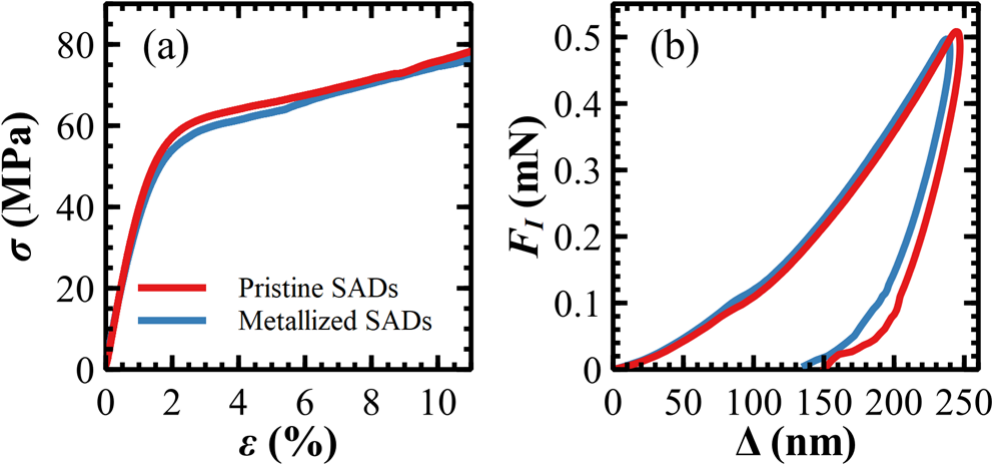
(a) Stress–strain curves before failure. (b) Nanoindentation curves, the average of more than 50 measurements. Data obtained for pristine (red) and metallized (blue) SADs.

For pristine SADs, the Young modulus *E*_TT‑P_ can be calculated from the elastic region as (3.6 ± 0.2) GPa, whereas the yield point occurs at stress σ_Y–P_ and strain ε_Y–P_ of (39.9 ± 1.2) MPa and (1.13 ± 0.07)%, respectively (Table 1). Then, the samples exhibit ductile behavior with a large plastic deformation region for a linear increase in the stress applied. This behavior ends when a rupture occurs in the central area of the sample. This proves that the specifically designed sample holder configuration is effective in reducing a stress concentration at the grip points.^62^ The failure force *F*_F–P_ is equal to (39.3 ± 3.4) N, corresponding to a stress σ_F–P_ of (78.5 ± 6.8) MPa and a strain ε_F–P_ and (11.9 ± 2.8)%, respectively.

**Table 1:** Tensile and Hardness Properties of pristine (P) and Metalized (M) SADs^a^

	*E*_TT_ (GPa)	σ_Y_ (MPa)	ε_Y_ (%)	*F*_F_ (N)	ε_F_ (%)	σ_F_ (MPa)	*E*_IT_ (GPa)	*H*_IT_ (MPa)
P	3.6 (0.2)	39.9 (1.2)	1.13 (0.07)	39.3 (3.4)	11.9 (2.8)	78.5 (6.8)	8.39 (0.41)	424 (35)
M	3.4 (0.3)	38.7(1.8)	1.13 (0.08)	52.8 (7.3)	14.0 (4.0)	84.2 (10.3)	8.71 (0.56)	387 (39)

a Absolute errors in brackets.

The stress–strain response of the metalized surface is similar to that of the pristine counterpart, confirming that the metallization process does not significantly alter the mechanical behavior of the SADs. Indeed, the failure values (σ_F‑M_ = (84.2 ± 10.3) MPa, ε_F‑M_ = (14 ± 4) %), yield (σ_Y‑M_ = (38.7 ± 1.8) MPa, ε_Y‑M_ = 1.13 ± 0.08%), and the elastic modulus (*E*_TT‑M_ = (3.4 ± 0.3) GPa) are equal to those obtained for pristine SADs, within the experimental errors. Among the mechanical parameters, solely the force at failure *F*_F‑M_ = (52.8 ± 7.3) N exhibited a higher value than that of the pristine SADs. This difference is most likely due to the increased thickness of the metallized SAD sample, which measures (125 ± 5) μm compared to (100 ± 2) μm for the pristine sample.

The results of the nanoindentation measurements are summarized in Table 1 and Figure 7b. The metallized surfaces exhibit an average hardness value *H*_IT_ lower than that for pristine samples. This can be deduced from the depth Δ*r*emaining after setting the force back to zero (Figure 7b), which is larger for the pristine sample. Conversely, the Young modulus *E*_IT_ of the metallized sample is about 4% larger, i.e., the beginning of the unloading curve is slightly steeper for the metallized surface. Such a difference is, however, within the respective standard deviations, which quantify the variations observed in different areas of the sample. Thus, both parameters can be considered to be nearly unchanged.

The tensile and instrumented indentation tests prove that the metallization process negligibly affects the mechanical properties of the SADs. The samples exhibit an anisotropic behavior of the Young modulus: the “in-plane” value measured by tensile tests is (3.5 ± 0.3) GPa while the “out-of-plane” value determined by nanoindentation is (8.55 ± 0.49) GPa. Both values agree with the literature,^63–65^ except in one case where it is lower, viz. (2.4 ± 0.2) GPa.^66^ The tensile strength σ_*F*_, i.e., the maximum stress that a material can bear before failing when it is allowed to be stretched or pulled, is ≈81 MPa, higher than those reported in the literature for SA: ≈34, ≈38, ≈60, and ≈70 MPa.^66–69^ Such differences can be ascribable to different M/G ratios and/or molar mass.

The metallized SADs present an average unstressed electrical resistance *R*_0_ of (184 ± 20) Ω. When subjected to tensile stress, the electrical resistance *R* exhibits a monotonical increase for increasing strain (see Figure 8a), characterized by two linear segments separated by a knee at the yield point. The change of the normalized resistance (*R*·*R*_0_^–1^) is less pronounced in the initial region: *R* increases by only (1.6 ± 0.4)% with respect to *R*_0_, compared to the plastic region where the increase is (62.7 ± 18)% before failure.

**Figure 8:**
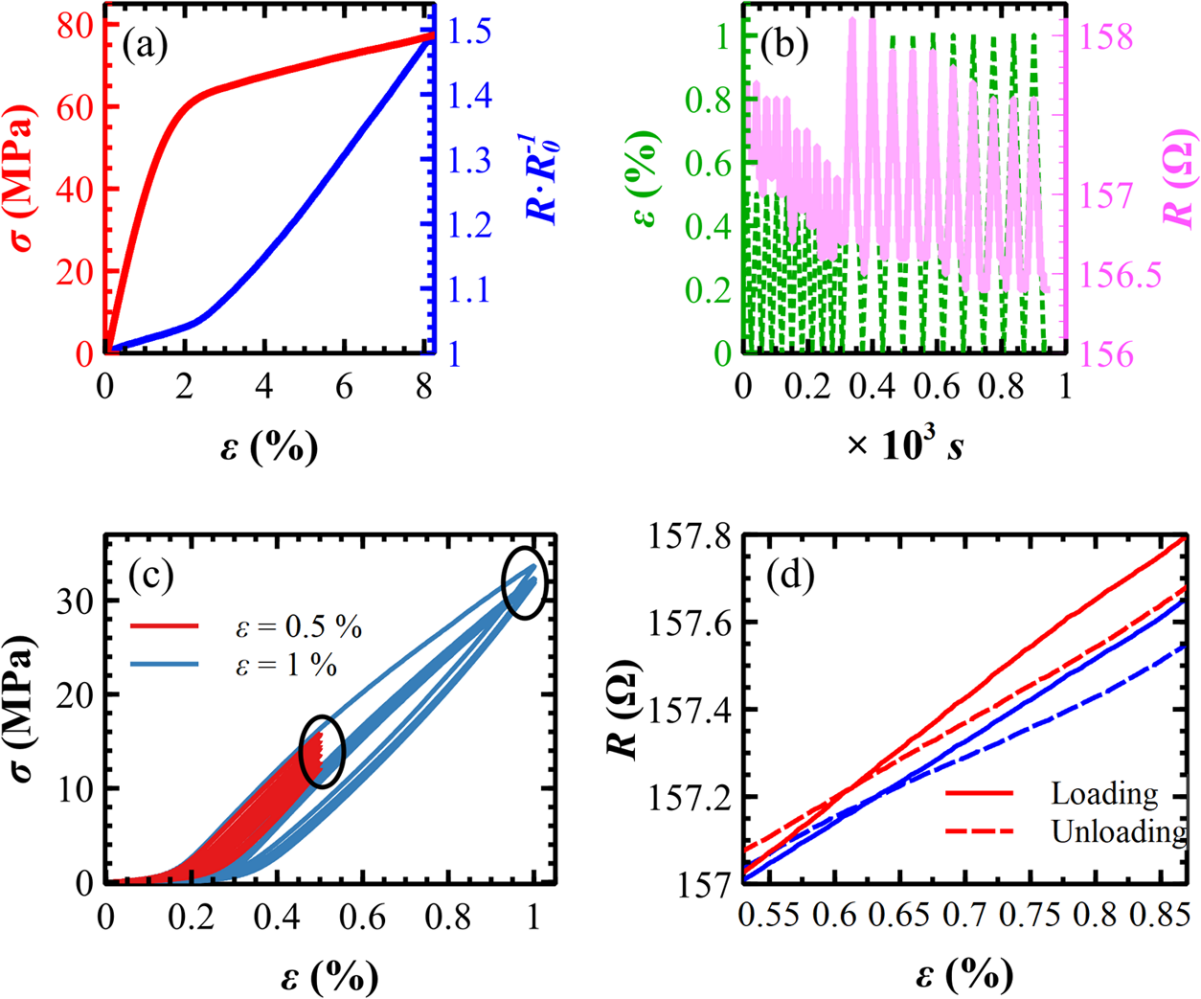
(a) Electrical resistance *R* normalized with respect to the unstressed value *R*_0_ (ε = 0) plotted vs strain ε. (b) Cyclic tests: ε and *R* plotted as a function of time *t*. (c) Stress–strain curves: 10 cyclic tests are plotted here; the black ellipses indicate the relaxation of the material. (d) Linear trends of *R* vs ε: Two consecutive loading and unloading curves are plotted for illustrative purposes.

The electro-mechanical measurements were necessarily performed in the elastic region; if the metallized SADs were plastically deformed, their initial lengths cannot be recovered and so *R*_0_. Cyclic tests of *R* vs ε, from unstressed (ε = 0) to a maximum strain ε_MAX_ of 0.5 and 1%, were performed for 10 times (dashed green lines of Figure 8b). The *R* curves are in phase with the strain patterns (pink lines) for both ε_MAX_ values. The *R* variations for all cycles are always significant, as the multimeter employed is very sensitive, 10 μΩ (LCR-meter LCR-6200, GW Instek, Montclair). Both strain cycles show that *R* drifts toward lower values (in a few cases, higher, not shown). This drift is continuous for ε_MAX_ = 0.5%, while it disappears after 7 cycles for ε_MAX_ = 1%. Such behavior can be interpreted using the stress–strain plot reported in Figure 8c.

As highlighted by one of the black ellipses, σ at ε_MAX_ = 0.5% exhibits a continuous lowering of its value for successive cycles, indicating that the material relaxes.^70^ On the other hand, σ at ε_MAX_ = 1% shows mostly overlapped curves after a few initial cycles, suggesting a minimized relaxation of the material. As empirically determined from these data, a suitable ε range to obtain a linear response of *R* is 0.53% < ε < 0.87%, after applying some strain cycles at ε_MAX_ = 0.5% to precondition the material. As visible in Figure 8d, *R* is linear in this range with different slopes for loading, ≈2.02 Ω·%^–1^ (continuous lines), and unloading, ≈1.64 Ω·%^–1^ (dashed lines), due to a small hysteresis between them.

Bending tests were then performed to correlate the radius of curvature (ROC) in the middle of the stripe with the maximum bending permitted to a metalized SAD without a loss of electrical conductivity. The samples used in this experiment were rectangular stripes cut from the metallized SADs. The thickness (≈0.15 mm) can be considered negligible with respect to the lateral dimensions (30 mm × 10 mm). According to ref 71, these samples can be modeled as paper stripes doubly clamped, and their mechanical bending can be described by minimizing the bending potential energy for a constant length. Simulated tests, using the experimental data for the clamp distance *L* and the maximum height *H*_max_ (see Figure 9a), provide results comparable to the experimental ones.^33,72^ As defined in ref 73, the ROC values are calculated at the middle of the stripe (*H* = *H*_max_, see Supporting Information). The electro-mechanical behavior can be investigated in two configurations, *viz*., the metallized side pointing up or down.

**Figure 9:**
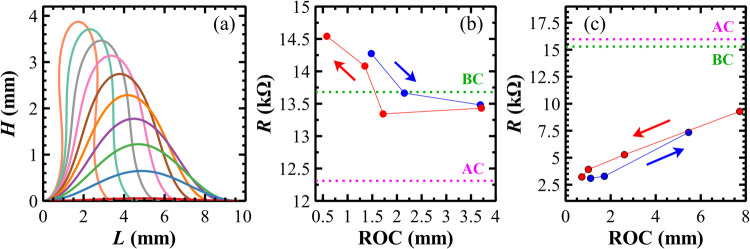
(a) Simulations of a stripe with the metallized side pointing up: for each curve, the *H*_max_ and *L* values are the measured ones. (b) Experimental resistance *R* data plotted vs ROC values (metallized side pointing up). The ROC values are extracted from the simulations (see Supporting Information). The red and blue arrows indicate forward and backward bending paths, respectively. Since an unstressed sample has ROC → ∞, the dashed green line indicates the *R* value ‘Before Compression’ (BC), while the dashed fuchsia line indicates the *R* value ‘After Compression’ (AC). (c) Experimental resistance *R* data plotted vs ROC values (metallized side pointing down). Since ROC → ∞ for an unstressed sample, (b, c) plots must be read from right to left. The relative experimental errors of the *R* data are negligible, 10^–4^–10^–5^%.

For a metallized side pointing up, an unstressed stripe has an *R* = 13.68 kΩ when ROC → ∞ (flat configuration), indicated by the dashed green line (BC) in Figure 9b. At the first bending with a ROC = 3.71 mm, corresponding to a *H*_max_ = 1.3 mm (green curve in Figure 9a), *R* slightly decreases to 13.43 kΩ and remains about constant up to a ROC = 1.72 mm. After that, *R* increases monotonically up to 14.54 kΩ for the lowest measured ROC = 0.58 mm, with an increase of about 6% from the unstressed value (Figure 9b). When the bending is reduced to the initial position (blue arrow in Figure 9b), *R* increases for comparable ROC values, showing hysteresis. After compression, the unstressed sample has a lower *R* value, indicated by the dashed fuchsia line marked (AC) in Figure 9b.

For a metallized side pointing down, an unstressed stripe has *R* = 15.29 kΩ when ROC → ∞ (flat configuration), as indicated by the dashed green line (BC) in Figure 9c. At the first bending with a ROC = 7.73 mm, corresponding to a *H*_max_ = 0.6 mm (blue curve in Figure 9a), *R* drops to 9.29 kΩ (≈40% less) and then it decreases linearly to 3.22 kΩ for the lowest measured ROC = 0.72 mm (red arrow in Figure 9c). When the bending is reduced to the initial position, *R* decreases for comparable ROC values with hysteresis, but the values coincide again at a ROC = 5.46 mm. After compression, the unstressed sample has a higher *R* value, indicated by the dashed fuchsia line marked (AC) in Figure 9c.

## IV Discussion

The subsurface region imaged in Figure 6a shows a transverse plane of the sample at ≈20 nm from the surface; Au clusters have various sizes and are homogeneously distributed, but they occupy a small portion of the surface with respect to the light gray region of the polymer. On the other hand, the transverse plane, few nanometers below the surface, imaged in Figure 5d by AFM shows a layer of Au clusters dense and compact. Such measurements suggest a cluster density decrease with increasing depth in the SA substrate.

This is confirmed by an additional TEM image of a cross-sectional slice in Figure 10a; the Au implanted layer is roughly composed of a darker region of ≈15 nm (light orange area in Figure 10a) where the clusters are denser and continuous, and a larger region of ≈80 nm thickness (light magenta area in Figure 10a) where the clusters are less dense and discontinuous. The former is consistent with subsurface regions imaged in Figure 5d, and the latter is consistent with that of Figure 6a. The sketch in Figure 10b summarizes the cluster density cross-sectional profile: within the first ≈5 nm, it is high, decreasing rapidly within ≈10 nm, after that Au clusters are less dense and more separated for the remaining ≈80 nm.

**Figure 10:**
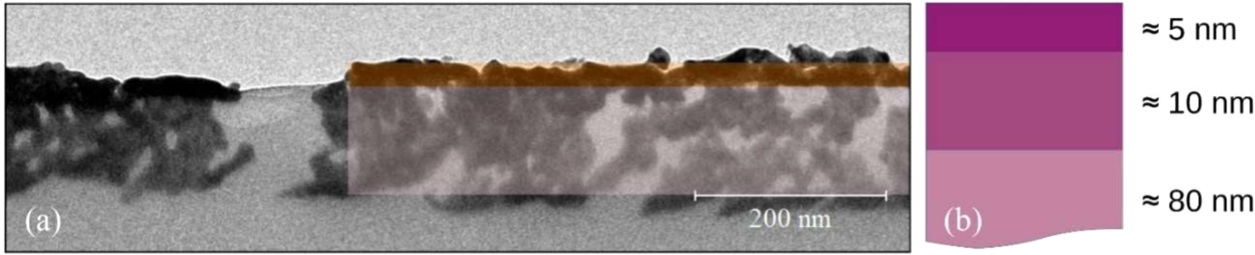
(a) Digital zoom of a bright field TEM image highlighting the two regions with a different density of Au clusters. (b) Sketch of cluster density: Purple colors are used to highlight regions with different densities at different depths.

Accordingly, the electrical current flows mainly within a subsuperficial region ≈15 nm thick, especially within a compact and ordered layer close to the surface (Figure 5d). Such a layer is so thin that it breaks even for a small bending, ROC = 3.71 mm, as proved by the increase of *R* in compression (metallized side pointing up). After further bending, this continuous ultrathin layer becomes composed of separated Au islands, as evident by the cracks visible in Figure 5d. At a moderate ROC (1.72 mm), the metallic regions are more separated, and *R* increases, and so on for further smaller ROC values. In compression tests with the metalized side pointing down, *R* largely decreases because the metallic regions are forced to move closer.

This picture, together with the presence of inhomogeneities due to sputtering deposition (see Supporting Information), indicates a tridimensional percolative conduction in the embedded metallic layer,^74^ that explains why *R* spans from a few hundred Ω to a few kΩ.

Elastic and plastic electro-mechanical behaviors are also part of the percolation framework of an Au/polymer composite. In the elastic regime, the polymeric portions of the composite move away and bring together the clusters during strain cyclic tests (see Figure 8b). In the plastic regime, the polymeric portions, once deformed, do not recover their original shape; however, the electrical conductivity is still nonzero up to the sample fracture: 3D percolation paths are active until the physical rupture of the sample.

## V Conclusions

Sodium alginate foils metallized by plasma sputtering with gold clusters were electromechanically investigated using tensile and hardness tests. Concurrently, morphological and structural characterization of the samples allowed, for the first time, the elucidation of the composition and distribution of metal clusters while also addressing several open questions reported in the literature concerning the incorporation of metallic clusters within polymeric matrices. Both pristine and metallized SA foils show an anisotropic Young modulus, making them more stretchable “in plane” with respect to “out of plane”. In the elastic regime, variations of the electrical resistance are conformal to the strain cycles. This result opens the possibility of using such a composite as a force sensor to determine an unknown strain imposed on the material. The good reproducibility of the electrical resistance variations in strain cyclic tests makes them robust candidates for the fabrication of strain sensors.

## Supplementary Material



## Notes

Calibration of Au sputtering: deposition rate and film thickness, Thread orientations on a pristine SAD, Au clusters deposited on Highly Oriented Pyrolytic Graphite, and Bending simulation of a stripe. (PDF)
