# Supplementary material for: Electro-Mechanical Properties of Metallized Sodium Alginate Foils at the Limit of the Electrical Conduction
Source: ACS Omega. 2025 Jul 18;10(29):32326–38. doi: 10.1021/acsomega.5c04447 (PMC12311676; doi:10.1021/acsomega.5c04447)
Supplement: Supplementary file 1 [file ao5c04447_si_001.pdf]

Supplementary Material

Electro-mechanical properties of metallized sodium alginate foils at the limit of the electrical conduction

Cristiano Albonetti, Carlo Gotti, Luca Pasquini, Nicola Gilli, Fabiola Liscio, Angela Longo, Stefano Chiodini, Franco Dinelli, Maria Letizia Focarete, Mirko Seri Monica Bertoldo, and Piera Maccagnani

1. Calibration of Au sputtering: deposition rate and film thickness

The deposition parameters used to metallize sodium alginate disks (SADs) were calibrated by depositing gold (Au) on Si wafers coated with native oxide. Based on previous studies,[1–3] the Au deposition was carried out by Ar^+ sputtering with a power of 20 W for 80, 160 and 300 s. The minimum time to obtain reproducible conductivity values for SADs is 160 s.

The largest thickness (deposition time of 300 s) was deposited on the Si wafer with a mask drilled with holes of 1.6 mm in diameter. This kind of sample is useful to verify the homogeneity of the film thickness *h* from the center of the wafer to its border. Film defects or dots scratches, gently produced with a metal tip, were used to evaluate *h* from the AFM topographic images. From the center to the border, there are ten dots (see inset in Figure S1). Step profiles were taken on the first, the fifth and ninth dots. Often the images are affected by a lateral inclination, due to tip-step convolution.[4] However, all programs for data analysis like Gwyddion (as visible in Figure S1) have tools to correct it and measure the step height *h*.[5]

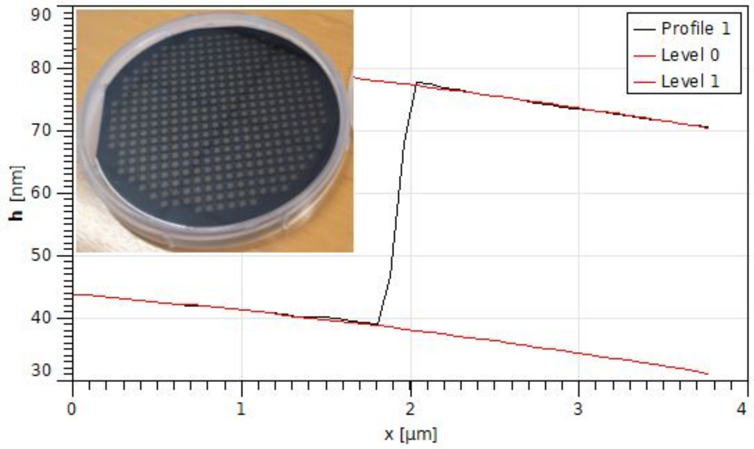

Figure S1: Illustrative procedure to fit a step with Gwyddion: two parallel polynomial curves (2nd degree) are used to identify top and bottom levels defining the step and correctly measure the step height *h*. Inset: picture of the Si wafer with Au dots.

As visible in Figure S2a, *h* decreases linearly from the center (defined as the inner circle with a diameter ≈ 3.2 mm including 4 dots) to the border, *viz*. the ninth dot that is ≈ 14.4 mm apart from the wafer border. Since the wafer is 4 inches in diameter, ≈ 102 mm, such a linear decrease occurs along ≈ 33.4 mm. Considering the first dot in the center, the fifth dot is ≈ 17.7 mm apart from it, while the ninth is at ≈ 33.4 mm. In the wafer center, *h* is equal to h_0 (see Figure S2a) but it decreases following the relationship $h = h_0 - 0.37 \cdot x$, where *x* (in mm) is the position along the wafer radius. The overall slope thus calculated is – (0.37 ± 0.03) $nm \cdot mm^{-1}$. Once the positional calibration is ended, the deposition rate $\mathcal{R}$ can be evaluated more accurately. As reported in the literature,[6,7] h_0 vs the deposition time *t* follows two linear behaviors: $\mathcal{R}$ at the early stage of the deposition (first 30 s in Ref. [6]) is relatively low, and after it accelerates.

As visible in Figure S2b, h_0 vs *t* is consistent with this trend. The linear behavior reported in the graph is the one with a higher $\mathcal{R}$ as it intercepts the *x*-axis at ≈ 40 s (arrow in Figure S2a). The $\mathcal{R}$ measured is (0.15 ± 0.01) $nm \cdot s^{-1}$, similar to the one reported in Ref. [6] 0.18 $nm \cdot s^{-1}$. The SADs investigated in this work were metallized for 160 s, that is the minimum time to make SADs conductive. Assuming a deposition time ≈ 30 s and a lower deposition rate $\mathcal{R}_L$, with a ratio $\mathcal{R} \cdot \mathcal{R}_L^{-1} = 2$ as reported in Ref. [6], h_0 is the sum of 2.25 nm for 30 s at $\mathcal{R}_L = 0.075$ $nm \cdot s^{-1}$ and (19.5 ± 1.3) nm for 130 s at $\mathcal{R} = 0.15$ $nm \cdot s^{-1}$. Thus, h_0 is (21.8 ± 1.3) nm.

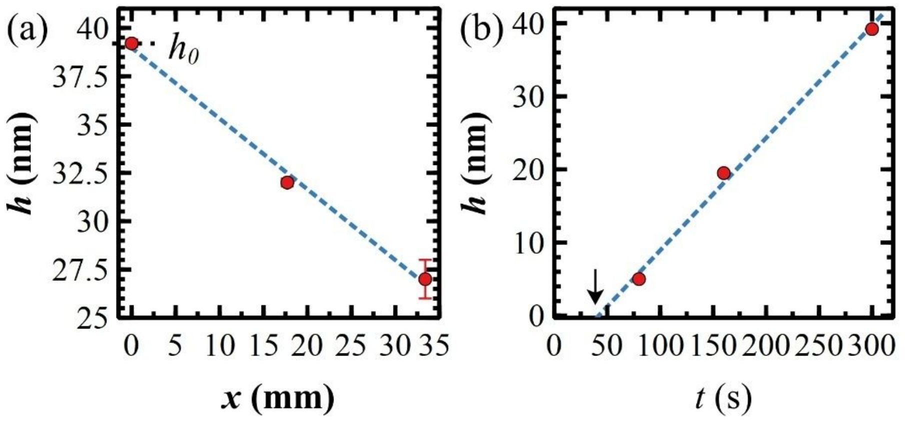

Figure S2: (a) Film thickness *h* vs the position on the wafer *x* from the wafer center (x = 0 mm). (b) *h* vs deposition time *t*, when the deposition rate $\mathcal{R}$ is faster.

2. Thread orientations on a pristine SAD

As shown in Figure 2, the SAD side in contact with the Petri dish presents a morphology characterized by the presence of linear threads. The orientation of these threads on different regions of a pristine SAD were investigated by fixing the fast scan orientation of AFM perpendicular to a given radius (see dotted red line in Figure S3). Five pieces of a pristine SAD were cut along the radius direction from the center to the disk border (regions 1, 2 and 3 in Figure S3) and along an internal circumference arc (regions 4 and 5). The images reported in the manuscript were taken from the center (missing rectangle in Figure S3 – Top).

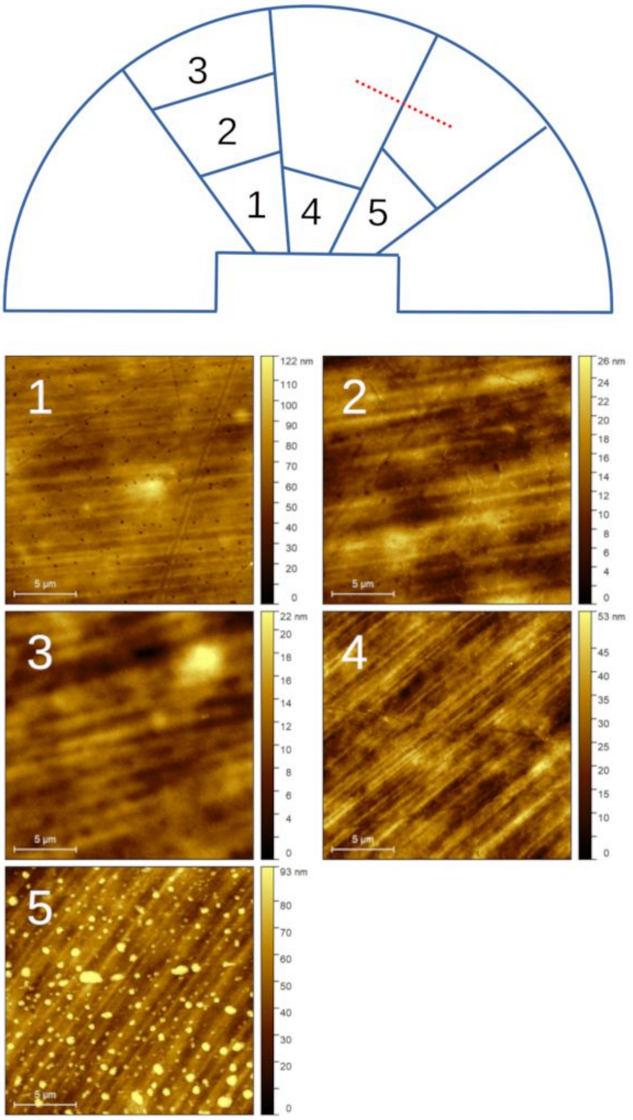

Figure S3: Top: scheme of the location where the various pieces analyzed were cut from. Bottom: AFM topographic images of various pieces.

As visible in Figure S3, the threads are present everywhere with a random orientation: quasi parallel to the scan direction (regions 1, 2 and 3), ≈ 45° (region 4) and ≈ 60° (region 5).

3. Au clusters deposited on Highly Oriented Pyrolytic Graphite

Au deposition was made on Highly Oriented Pyrolytic Graphite (HOPG) using the experimental conditions adopted for the metallization of SADs but with a deposition time of only 5 s. In Figure S4a, the Au clusters are visible as bright dots that cover only about 0.3 % of the entire surface. Inspecting the HOPG surface at a larger magnification (Figure S4b), one can notice that the Ar^+ bombardment produces ripples that increase the surface roughness to ≈ 3 Å, as already observed in the literature.[8]

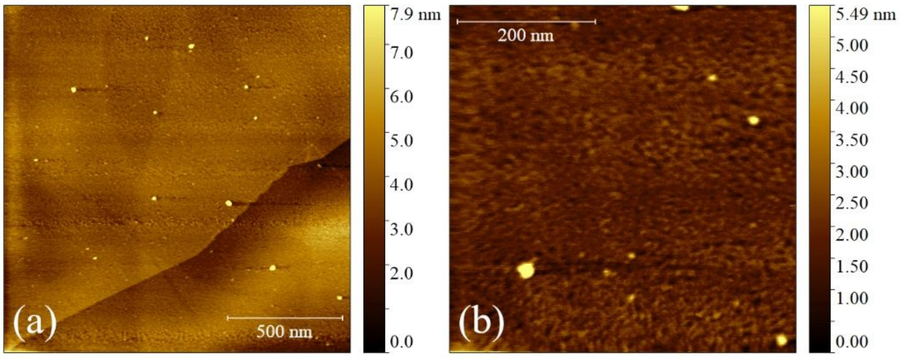

Figure S4: (a) A representative example of AFM topographic images adopted to analyze the size distribution of the Au clusters. Topographic profiles on each cluster were used to measure the height, that corresponds to their diameter under the assumption of a spherical shape for all the clusters. (b) Higher magnification image of the surface highlighting ripples produced by the Ar^+ bombardment.

4. Bending simulation of a stripe

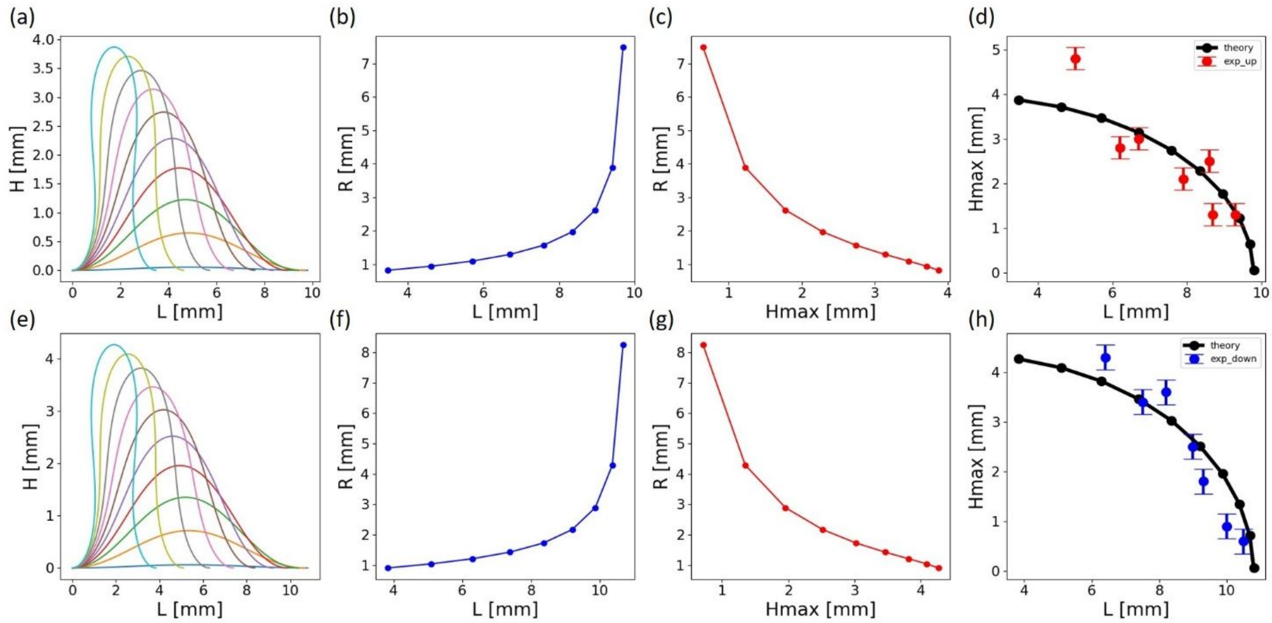

Figure S5: Simulations and experimental data in the case of bending a stripe with the metallized side pointing up. (a) Simulated stripe shape for 10 different positions (*L* values) of the right clamp with reference to a fixed clamp on the left at L = 0 (up case). (b) Radius of curvature (ROC) at the peak of the stripe as a function of the lateral stripe extension *L*. (c) ROC at the maximum stripe height *Hmax* in the middle of the stripe. (d) *Hmax* vs. *L*: simulated data, obtained by the model of Ref. [9] (black dots), and experimental results (red dots). (e-h) Simulations and experimental data in the case of bending a stripe with the metallized side pointing down.

Bibliography

(1) Summonte, C.; Maccagnani, P.; Desalvo, A.; Bolognini, G.; Ortolani, L.; Sanmartin, M.; Capelli, R.; Bertoldo, M.; Dinelli, F. Gold Nanoparticles on Sodium Alginate: Simulation of Optical Properties. In *20th Italian National Conference on Photonic Technologies (Fotonica 2018)*; 2018; pp 1–4. https://doi.org/10.1049/cp.2018.1637.

(2) Summonte, C.; Maccagnani, P.; Maurizi, A.; Pizzochero, G.; Bolognini, G. Simulation of the Optical Properties of Gold Nanoparticles on Sodium Alginate. *EPJ Web Conf.* **2021**, *255*.

(3) Summonte, C.; Maurizi, A.; Rizzoli, R.; Tamarri, F.; Bertoldo, M.; Bolognini, G.; Maccagnani, P. Experimental Analysis and Simulation of the Optical Properties of Gold Nano-Particles on Sodium Alginate. *Opt. Mater. Express* **2022**, *12* (11), 4456–4470. https://doi.org/10.1364/OME.465985.

(4) Zhao, X.; Vorburger, T.; Fu, J.; Song, J.-F.; Nguyen, C. A Model for Step Height, Edge Slope and Linewidth Measurements Using AFM; Proceedings of American Institute of Physics International Conference on Characterization and Metrology: 2003 International Conference (AIP Conference Proc. 683), edited by D.G. Seiler, et al. (Amer. Inst. Phys, Woodbury NY), Austin, TX, 2003.

(5) Nečas, D.; Klapetek, P. Gwyddion: An Open-Source Software for SPM Data Analysis. *Cent. Eur. J. Phys.* **2012**, *10* (1), 181–188. https://doi.org/10.2478/s11534-011-0096-2.

(6) Švorčík, V.; Slepička, P.; Švorčíková, J.; Špírková, M.; Zehentner, J.; Hnatowicz, V. Characterization of Evaporated and Sputtered Thin Au Layers on Poly(Ethylene

Terephtalate). *J. Appl. Polym. Sci.* **2006**, *99* (4), 1698–1704. https://doi.org/10.1002/app.22666.

(7) Allen, T. D.; Simmens, S. C. Conversion of Vacuum Coating Units for Sputter Coating. *Micron (1969)* **1976**, *7* (2), 141–144. https://doi.org/https://doi.org/10.1016/0047-7206(76)90057-1.

(8) Hohner, C.; Kettner, M.; Stumm, C.; Schuschke, C.; Schwarz, M.; Libuda, J. Pt–Ga Model SCALMS on Modified HOPG: Growth and Adsorption Properties. *Top. Catal.* **2019**, *62* (12), 849–858. https://doi.org/10.1007/s11244-019-01167-0.

(9) Batista, A. A. The Mechanics of Bending a Strip of Paper. *Eur. J. Phys.* **2020**, *41* (6), 65009. https://doi.org/10.1088/1361-6404/ab9c8e.
